# Importance of Ecological Factors and Colony Handling for Optimizing Health Status of Apiaries in Mediterranean Ecosystems

**DOI:** 10.1371/journal.pone.0164205

**Published:** 2016-10-11

**Authors:** Irene Asensio, Marina Vicente-Rubiano, María Jesús Muñoz, Eduardo Fernández-Carrión, José Manuel Sánchez-Vizcaíno, Matilde Carballo

**Affiliations:** 1 Epidemiology & Environmental Health Department, Animal Health Research Center (CISA-INIA), Madrid, Spain; 2 VISAVET, Faculty of Veterinary Science, Universidad Complutense de Madrid (UCM), Madrid, Spain; 3 Animal Health Department, Faculty of Veterinary Science, Universidad Complutense de Madrid (UCM), Madrid, Spain; Sichuan University, CHINA

## Abstract

We analyzed six apiaries in several natural environments with a Mediterranean ecosystem in Madrid, central Spain, in order to understand how landscape and management characteristics may influence apiary health and bee production in the long term. We focused on five criteria (habitat quality, landscape heterogeneity, climate, management and health), as well as 30 subcriteria, and we used the analytic hierarchy process (AHP) to rank them according to relevance. Habitat quality proved to have the highest relevance, followed by beehive management. Within habitat quality, the following subcriteria proved to be most relevant: orographic diversity, elevation range and important plant species located 1.5 km from the apiary. The most important subcriteria under beehive management were honey production, movement of the apiary to a location with a higher altitude and wax renewal. Temperature was the most important subcriterion under climate, while pathogen and *Varroa* loads were the most significant under health. Two of the six apiaries showed the best values in the AHP analysis and showed annual honey production of 70 and 28 kg/colony. This high productivity was due primarily to high elevation range and high orographic diversity, which favored high habitat quality. In addition, one of these apiaries showed the best value for beehive management, while the other showed the best value for health, reflected in the low pathogen load and low average number of viruses. These results highlight the importance of environmental factors and good sanitary practices to maximize apiary health and honey productivity.

## Introduction

Recent considerations about possible environmental factors contributing to the global decline in bee populations have implicated an array of causes, among which pests, pathogens, pesticides, nutrition and management have become the most important [1–4]. Poor nutrition strongly influences honey bee immunocompetence [5] and therefore longevity, physiology and resistance or tolerance to disease [6]. Long-term consumption of a polyfloral diet can safeguard colony survival [7,8] and help bees remain healthy despite the numerous stresses to which they are exposed, including pest treatments, transport, honey extraction, loss of honey reserves in the colony, wax re-use, pollination in monocultures, presence of nearby apiaries, and overcrowding in the apiary. Adding to these stresses is the likelihood of spatiotemporal shortages of nutrients due to low food availability, nutrient deficiency in diet, low species richness, shortening of flowering time due to climate conditions, loss of habitat, land cover changes, agricultural intensification and lack of pollen and nectar resources [3]. Land-use change, in particular, has caused extensive loss of nutritional resources in the landscape, making it a major contributor to the global decline in honey bee populations [4, 9, 10].

Pathogens may also play a central role in honey bee population decline. Viruses are prevalent in apiaries around the world, and in some cases they have been associated with colony mortality [11–16]. However, some pathogens associated with colony mortality, such as bee viruses, usually persist in colonies at a low level in balance with the host without causing apparent symptoms in the individual or the colony, often referred to as ‘covert’ infection. Under certain conditions, viral replication is activated and the infection can become ‘overt’, leading to obvious symptoms [17]. This is true also of non-viral pathogens. *Nosema ceranae*, a gut microsporidium associated with colony collapse [18], has been observed in healthy colonies in asymptomatic infections [19]. It appears that the regenerative ability of the host digestive epithelium supports a certain level of *N*. *ceranae* multiplication, and that when the balance between pathogen and host is lost, symptoms can become overt. Monitoring these pathogens may serve as an indicator of colony health status and may predict colony strength and colony collapse [20–22].

Some factors inside and outside the colony can create a situation of stress. These factors can play a key role in maintaining or disturbing the pathogen-host balance and therefore triggering the passage from covert to overt infection. One of these factors is *Varroa destructor* infection; this pathogen harms individual bees by feeding on their hemolymph, and it harms the colony by vectoring several bee viruses and by triggering immunosuppression [23, 24]. Poor nutrition can increase colony susceptibility to bee viruses, as shown in a study of caged bees with increased levels of deformed wing virus (DWV) [25], and it can reduce colony tolerance to *N*. *ceranae* [6]. Other important stressors are those related to the exposure of colonies to phytosanitary treatments. Wax is an organic matrix that accumulates the residues from both internal treatments to control *Varroa* and from external pesticides brought into the colony as a result of foraging [26–28]. Pesticides can deplete the immune system [29, 30] allowing pathogens to replicate and have a negative effect in the colony (reviewed in [31]). For example, pesticide exposure has been associated with increased levels of *N*. *ceranae* [32] and with immunosuppression that promotes viral replication [29]. Adverse climate conditions can strongly influence honey bee activity and resistance to pathogens [33]. In addition, temperature influences the *Nosema* biological cycle [34]; the greater temperature resistance of *N*. *ceranae* compared to *N*. *apis* may facilitate its persistence in honey bee colonies around the world.

The complex multifactorial processes contributing to bee health and production are difficult to integrate in a model that would allow us to understand how factors related to the environment, production management and health, as well as their interactions, influence the productivity of a given apiary in the medium or long term. In order to accomplish this, the many relevant factors should be weighted based on relevance and combined appropriately into integrated indicators. In the present study we analyzed six apiaries in different Mediterranean environments using geographic information systems (GIS) and a multi-criteria decision analysis MCDA (analytic hierarchy process (AHP)) to identify the landscape and management factors most relevant to apiary health and bee production in the long term, allowing comparisons among all factors. GIS is useful to analyze spatial data, map relevant habitat features and finding areas where combinations of these features may worsen the conditions for the species of interest [35].

## Material and Methods

### Study area

**Fig 1:**
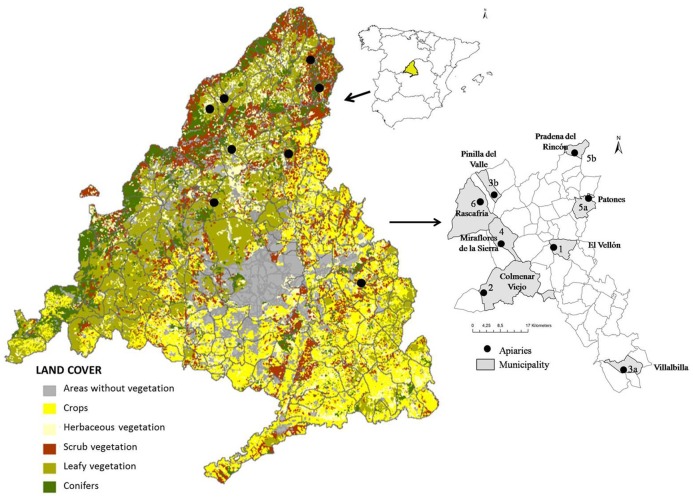
Location of apiaries in the study area and land cover types.

Six apiaries of *Apis mellifera iberiensis*, a non-endangered nor protected bee species, dedicated to honey production were studied (numbered 1–6). The apiaries were located in the northeast part of the Community of Madrid (Fig 1).

These apiaries were selected to represent a range of environments in order to capture natural variations in honey bee survival. Apiaries 1, 2, 4 and 6 were static, remaining in the same location all year round. Apiaries 3 and 5 were transhumant, changing from one location (3a, 5a) to another at a higher altitude (3b, 5b) to take advantage of different nutritional resources in late spring. Permission to visit the apiaries, record data and take samples was obtained directly from beekeepers and no specific permits from public authorities were required to perform these activities. GPS coordinates were recorded for each apiary location under confidentiality agreement, so location information is publicly available only at the municipality level (Fig 1).

### Factors analyzed

We considered 29 factors to characterize apiaries, belonging to five different categories: a) habitat quality, b) landscape heterogeneity, c) weather conditions, d) beehive management and e) health.

#### a) Characterization of habitat quality

Habitat quality was assessed over a 7.06 km^2^ territory extending to a radius of 1,500 m around each apiary based on GPS coordinates.

Habitat quality was assessed in terms of 9 factors: Number of land cover types (H1); Number of important vegetable species for bees (H2); Harvestable area unfragmented by infrastructures (H3); Distance to permanent watercourses (H4); Distance to roads (H5); Distance to power lines and antennas (H6); Elevation range (H7); Orographic diversity (slope surface) (H8) and Crop surface (H9). These factors were defined through a literature review, surveys to beekeepers and maps of the study area: the forestry map of the Community of Madrid [36] and the topographical map of Spain [37] using GIS tools (ArcGIS 10, ESRI). GIS was used to visualize data and to develop a spatial analysis of each study area.

In the case of habitat quality we collected supplementary information about 9 additional apiaries, also located in the Community of Madrid, to obtain a more precise calculation of the habitat quality factors. The factor concerning to number of land cover types (H1) was evaluated using the forestry map, that describes 81 land cover types, 74 of which correspond to land cover vegetation (birches, oaks, heathers, kermes oaks, junipers, beeches and conifers, among others). It was felt that more than 10 land cover types was the best situation for this factor. Therefore, the number of important vegetable species for bees in the study area (H2) was identified through an exhaustive literature search (floristic catalogues [38] among others), field work, surveys to beekeepers and spatial visualization using the forestry map [35]. Composition of each species (i. e. crude protein (%), lipids and nutritional value, among others) was used in order to classify factor H3. It was considered that the availability of > 12 important vegetable species presented in each area supposed the most favorable condition for bees (S1 Table).

As for fragmentation of the study area by infrastructures (H3), the absence of major roads, industrial estates, large bodies of water or urban areas around the study area was considered the best situation. Also, considering the distance to rivers and streams (H4), apiaries closed to those (≤ 50 m) were considered the best areas for bees (S1 Table).

Regarding distance to roads (H5) and distance to power lines and antennas (H6), the absence of primary and secondary roads and high and low voltage lines, repeater and antennas whitin 1500 m of each apiary were considered the best situations (S1 Table). Both factors were evaluated through the topographical map.

Elevation range (H7) was calculated through the topographical map [37], considering the contour lines around each apiary and evaluating the maximum and minimum elevations in each area. The difference between both elevations was used to calculate the elevation range (S1 Table). The higher elevation range in all the studied areas (540 m) was regarded as the most favorable condition for bees.

Orographic diversity (H8) was calculated as the slope surface through the contour lines. Slope ranges were defined in each study area, considering slopes < 5° as the worst value and slopes above to 10° is the best situation. Finally, crop surface (H9) was evaluated using the forestry map. The absence of crop surface was considered the best situation for this factor (S1 and S2 Tables).

Data obtained were normalized to convert heterogeneous factors based on various measurement scales to a common scale. The min-max normalizer linearly rescales every factor to the [0,1] interval. The values were transformed using the following formula: [x- min (x)]/[max (x)-min(x)], providing a theoretical value for each apiary. In the case of the non-static apiaries (3(3a, 3b) and 5 (5a, 5b)), the average of the normalized values obtained became the single value for each apiary.

#### b) Characterization of landscape heterogeneity

Suitability of landscape composition and configuration were assessed using the following factors: Total edge (L1), Mean patch size (L2), Number of patches (L3) and Patch size standard deviation (L4). These factors were studied through landscape structural analysis as described [39] using the ArcGIS extension "Patch Analyst" over an area of 7,06 km^2^ extending to a radius of 1,5 km around each apiary with the forestry map of the Community of Madrid [36]. Total edge (L1) refers to the sum of perimeters of all patches within the total area. Mean patch size (L2) refers to all patches within the total area. Number of patches (L3) refers to total number of patches in the studied area. Patch size standard deviation (L4) refers to the patch size variability. High values of each factor indicate optimal situation for an apiary (S3 Table).

Data obtained were normalized to convert heterogeneous factors based on various measurement scales to a common scale. The values were transformed using the normalized min-max, and rescaled the [0, 1] interval.

#### c) Characterization of weather conditions

A literature review was carried out to establish the influence of temperature, precipitations and wind speed over the bee activity [40–50] (S4 Table). In addition, climatic data were collected from April 1 to October 31, 2014 (214 days) at four meteorological stations belonging to the Spanish Meteorological Agency [51]. Climate data from the nearest meteorological station were taken into account for each apiary. The stations were located less than 25 km away from the apiaries at an altitude within 100 m of the apiaries. Given these data collected, 3 factors were assessed: average maximum temperature for each month (C1), number of days with a wind speed higher than 30 km^2^ (C2) and number of consecutive days without precipitation (C3). These values were also normalized.

#### d) Characterization beehive management

Information about apiary characteristics and beehive management was collected through surveys of beekeepers at the apiaries (S5 and S6 Tables). Data were codified in nine factors: honey production (M1), type of honey (M2), treatment used against *Varroa* (M3), number of treatments (M4), risk of treatment (M5), renewal of wax (M6), origin of wax (M7), movement of the apiary to another location (M8) and presence of extensive livestock in the natural area (M9). The best and the worst situation of each factor were determined and then ranges were defined. Annual honey production (M1) across the six apiaries ranged from 15 kg/colony to 70 kg/colony. This last value is considered an excellent production.

In addition, we considered other characteristics to define the best situation for apiaries, i. e. a greater number of honey produced types (M2), the use of ethereal oils or organic acids against *varroa* instead of pesticides (M3) or a reduced number of pesticide treatments throughout the year (M4) (S5 Table).

Also, the hazard posed by anti-*Varroa* treatments (M5) was assessed by taking into account the bee-toxicity data, degradation products and their ability to accumulate in beeswax of the treatment agents used as well as the frequency of wax renewal. Toxicity data on the main active substances were taken from the Ecotox Database of the US Environmental Protection Agency, the Hazardous Substance Data Bank (TOXNET database) and the research literature. The lipophilic capacity of the substances was used to determine their potential to accumulate in beeswax. Each parameter was assigned a score from 0 to 3 (S6 Table). The higher the score was, the more likely it was that the substance would accumulate in wax. An adequate wax renewal (M6) was defined as wax change in all frames of every hive every 2–3 years.

In addition, a high wax quality (light colour) with an adequate origin (i.e. traceable, good manufacturing practices, etc.) (M7), a move of the apiary to take advantage of nutritional resources (M8) and absence of extensive livestock in the surroundings of the apiary (M9) were considered excellent conditions (S5 Table).

Results were also normalized.

#### e) Characterization of health

Processing and molecular analysis of honey bee samples. The study period was from May to November 2014. Honey bees were collected from each apiary twice during the beekeeping season: May-June 2014, referred to hereafter as “spring-summer sampling”; and August-September 2014, referred to as “summer-autumn sampling”. During sampling, adult bees (foragers) and brood were taken from each colony, refrigerated during transport and frozen at -80°C. Ten whole bees or brood (larvae and pupae) were homogenized separately in 2 ml PBS (pH 7.2) using a mortar and pestle. Adult and brood samples from each colony were analyzed for the presence and load of six viruses and one microsporidium (collectively referred to hereafter as “pathogens”) by amplification of pathogen-specific nucleic acid, followed by absolute quantification. The following pathogens were analyzed: acute bee paralysis virus (ABPV), Israeli acute paralysis virus (IAPV), Kashmir bee virus (KBV), sacbrood bee virus (SBV), deformed wing virus (DWV), black queen cell virus (BQCV) and *N*. *ceranae*. Viral RNA was extracted from 150 μl of homogenate using the RNA II kit (Macherey-Nagel) according to the manufacturer’s instructions. *N*. *ceranae* DNA was extracted by crushing frozen pellets of the homogenates and using the DNA Isolation kit (Roche) according to the manufacturer’s instructions. Quantitative polymerase chain reaction (qPCR) with or without prior reverse transcription (RT) were carried out using SYBR Green dye, primers and PCR conditions previously described [52–56].

During both samplings, each colony was carefully inspected for the presence of *Varroa destructor* by opening a small piece of the brood comb and searching for the presence of the mite. Though we were unable to collect data on *Varroa* load at the start of the study period, beekeepers at all apiaries indicated that colonies co-existed with the mite. *Varroa* was quantified in at least five colonies per apiary in October-November 2014 using the quantification-after-treatment method [57].

Pathogen presence and load were used as indicators of colony health status. Specifically, three factors related to *N*. *ceranae* and viruses were evaluated in each apiary during both samplings: pathogen load (He1), pathogen frequency (He2) and pathogen coinfection (He3). To measure He1, samples of adults and brood were analyzed separately for each colony. The greater pathogen load was log_10_-transformed and defined as the pathogen load for that colony and pathogen. These pathogen loads were normalized and the mean value for all pathogens was taken to be He1 for each apiary and sampling. He2 was calculated for each sampling by averaging the frequencies of colonies per apiary that were positive for each pathogen. He3 was calculated for each sampling by averaging the numbers of pathogens present in the same apiary. Colonies in which a pathogen was not detected in either adults or brood were considered negative for that pathogen. In addition to these three pathogen-related factors, *Varroa* load (He4) was also evaluated in each apiary during both samplings. He4 was calculated as the number of mites counted per colony after acaricide treatment.

### Multi-criteria decision analysis: Analytic hierarchy process (AHP)

There are several decision processes to compare alternatives that are different in their potential impact or outcome, to synthesize information: ad hoc decision-making, comparative risk assessment (CRA) or multi-criteria decision analysis (MCDA), portfolio decision analysis (PDA) among others [58, 59] can be useful to carry out this comparison.

MCDA establishes priorities among the actions or factors considered, based on a hierarchy of objectives and criteria, each weighted, on the basis of value judgments and technical relevance to fixed goals [35]. Complex MCDA methods are multi-attribute utility theory (MAUT), multi-attribute value theory (MAVT) and analytic hierarchy process (AHP) [58].

Selecting an appropriate MCDA method depends on the context. It is based on whether or not there are multiple objectives. Therefore, the method can be decided depending on the number of alternatives considered in the study. A compensatory method was developed in the present study, since there were no multiple objectives and there was no large number of alternatives. First, a criteria weighing method (AHP) was carried out to compare pair-wise criteria, and then a compensatory aggregation method (weighted linear combination) was also carried out to normalize criterion scores to enable comparison of performance on a common scale [35].

AHP was used to perform multi-criteria decision analysis in order to categorize apiaries from best to worst based on the values obtained for each of the measured factors. Normalized values obtained in each evaluated factor were used to carry out AHP. A hierarchical diagram was developed in which the target was on the first level, criteria on the second, sub-criteria on the third, and finally the alternatives available for achieving the target. Criteria and sub-criteria were defined based on the following factors: relative to Habitat quality (H1, H2, H3, H4, H5, H6, H7, H8 and H9), relative to Landscape heterogeneity (L1, L2, L3 and L4), relative to Climate conditions (C1, C2 and C3), relative to Beehive management (M1, M2, M3, M4, M5, M6, M7, M8 and M9) and relative to Health (He1, He2, He3 and He4). The best alternative among all possibilities (6 apiaries) was obtained.

Pair-wise comparisons were performed among factors on the same level. Values were assigned to each factor relative to the other factors according to the following Likert scale: 1, equally important; 3, moderately important; 5, strongly important; 7, very strongly important; or 9, extremely important. Intermediate values (2, 4, 6, 8) were assigned in those cases where the decision was doubtful. Judgment matrices were developed according to AHP principles [60]. Subsequently, the same Likert scale was used to perform comparisons of the alternatives with respect to each sub-criterion. Then, a weighted linear method was also carried out to normalize criterion scores. A normalized matrix was obtained from each of the matrices, and priority vectors were estimated for each combination. These vectors were multiplied by each other to obtain the priority vector of each alternative with respect to the target; this vector was assigned a value between 0 and 1.

The consistency of judgments was checked after obtaining the final priority vector. To do this, we calculated the Consistency Index (CI = (λ_max_-n) / (n-1)) and compared it with the Random Consistency Index (RCI = CI / IA).

## Results and Discussion

The apiaries were located in the northeast part of Madrid, in a relatively intact natural Mediterranean ecosystem comprised predominantly of oaks in open woods (Apiaries 1, 2, 3b) and meadows (5). The ecosystem contained abundant pollen- and nectar-rich plants such as lavender, rockrose, thyme, rosemary, and heather. Apiaries 4 and 6 were located in humid and shady areas with abundant oaks and ash groves. Apiary 3a was located in an area with Mediterranean scrub and olive groves.

**Fig 2:**
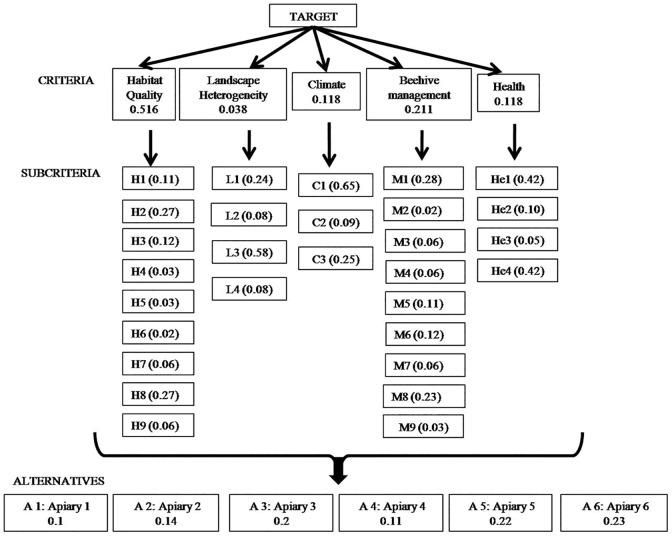
Analytic hierarchy process (AHP) to evaluate six apiaries in several natural environments. Development of criteria, subcriteria and alternatives to achieve the target, *i*. *e*. define the best alternative among possible. Numbers indicate relative weights of each criterion, subcriterion and alternatives.

**Fig 3:**
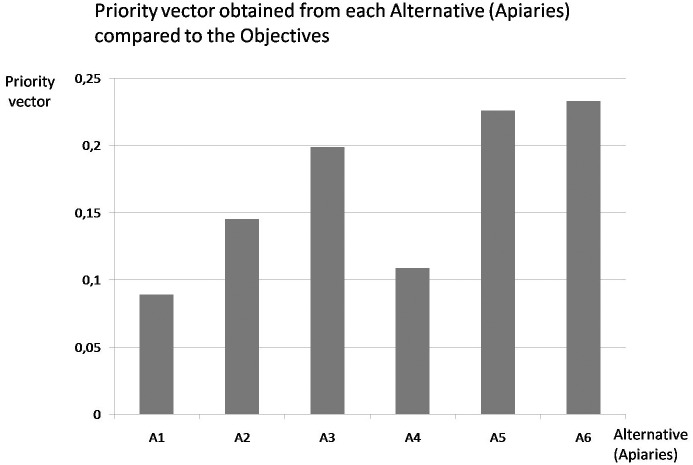
Results of the analytic hierarchy process (AHP).

AHP was used to generate 35 matrices: 29 matrices of alternatives with respect to sub-criteria, 5 matrices of sub-criteria with respect to criteria and 1 matrix of criteria with respect to the target (Figs 2 and 3 and Table 1).

**Table 1:** Development and results of the analytic hierarchy process (AHP).

Priority vector obtained from each Alternative (Apiaries) compared to the Criteria						**X**	Priority vector obtained from each Criterion compared to the Objectives		**=**	Priority vector obtained from each Alternative (Apiaries) compared to the Objectives
Apiary	Habitat Quality	Landscape Heterogeneity	Climate	Management	Health			Criteria		
A1	0.07	0.09	0.10	0.08	0.14		Habitat Quality	0.52		
A2	0.22	0.09	0.10	0.06	0.03		Landscape Heterogeneity	0.04		
A3	0.09	0.19	0.42	0.27	0.27					
A4	0.09	0.42	0.10	0.10	0.06		Climate	0.12		
A5	0.19	0.10	0.25	0.37	0.11		Management	0.21		
A6	0.31	0.09	0.03	0.09	0.38		Health	0.12		

In this analysis, habitat quality emerged as the most important criterion for evaluating apiaries (Table 1). Based on the priority vector obtained from each Alternative compared to the Criteria, the most important subcriteria in the evaluation were Important vegetable species for bees (H2), Orographic diversity (H8), Number of patches (L3), Temperature (C1), Honey production (M1), Colony movement (M8), *Varroa* load (He4) and Pathogen load (He1) (Fig 2). Based on the priority vector obtained from each Alternative compared to the Objective, Apiary 6 obtained the highest quality value, followed by Apiaries 5 and 3 (Fig 3). All resulting matrices were considered consistent because their RC values were lower than the maximal RC percentage (RC ≤ 0.10).

Habitat quality based on 9 factors was good at all the apiary locations (Table 1). Vegetation was abundant at 6 of the 8 locations, and 7 apiaries were located within 300 m of watercourses. On the other hand, 6 apiaries were also near roads. Three of the locations were below 300 m above sea level, and 5 locations featured low orographic diversity, with <5 km^2^ of surface area having a slope >5°. Infrastructures fragmented the territory at five locations (1, 4, 6, 3b, 5a). These infrastructures, which in our study area included main roads, urban areas and large reservoirs, divide habitats into smaller parcels, reducing the biodiversity and natural habitat available [4, 61–63].

AHP analysis assigned the lowest habitat quality (0.07) to Apiary 1. Several factors were responsible: nearby presence of a built-up area, presence of power lines 690 m from the apiary, and low elevation range (190 m). The low elevation range leads to homogeneous vegetation, with a reduced number of patches (21), reflected in its low landscape heterogeneity (0.09) [64]. Greater elevation variability in an area means the possibility of a larger number of habitats [65, 66]. As a result, many studies have used elevation range as an indicator of landscape heterogeneity [67, 68]. Apiary 1 had an intermediate health value (0.14). The He1 value was 0.45 at both samplings, though increases in the loads of *N*. *ceranae* and DWV were detected. In addition, He2 and He3 worsened from the first to second sampling. The increase of DWV load following the *Varroa* trend along the period of study is in line with previous work [69, 70]. Depopulation and winter mortality has also been attributed to this *Varroa*-DWV interaction [16, 20, 71], and we observed a slight increase in varroosis symptoms and a population reduction at the second sampling. Ascospherosis was observed in both samplings, which is probably related to the stress produced by high *Varroa* loads [72, 73].

The location of Apiary 2 showed high habitat quality (0.22), likely reflecting the high number of important vegetable species for bees (H2), even though the low elevation range (240 m) reduces the potential of vegetation succession. Indeed, like the location of Apiary A1, the location of Apiary 2 showed a relatively small number of patches (30) and low landscape heterogeneity (0.09). The health value of location 2 (0.03) was the lowest of all apiaries, reflecting the fact that He1, He2 and He3 increased from the first to second sampling. This deterioration in health status is consistent with a high *Varroa* load (mean of 1,319 mites per colony) and the presence of varroosis symptoms and deformed wings in all colonies sampled at the second sampling. The high *Varroa* load by itself may even explain the increased pathogen load, since the mite can immunosuppress honey bees [23] as well as serve as a vector of bee viruses, especially DWV, which replicates inside the mite [74, 75]. During the second sampling all colonies studied presented DWV loads above 10^6^ GEC/bee as well as overt symptomatology. These characteristics may predispose the colony to collapse during overwintering [15, 16]. Another factor contributing to the poor health state at Apiaries 1 and 2 may be climate: temperatures in July 2014 were unusually warm (>35°C) and the summer featured long periods without precipitation (45 days). This may have shortened the period of bloom around Apiaries 1 and 2 at these altitudes, which likely means that the bee populations did not have sufficient nutritional resources and were therefore more susceptible to infection [4]. This may also explain why these two apiaries showed the lowest annual honey production (15 kg/colony) of all six apiaries. Several studies have established a relationship between poor weather conditions and poor honey production [76, 77]. Another factor that may explain the poor health status at Apiaries 1 and 2 is the pesticides used to control *Varroa destructor*. Apiary 1 used Checkmite (coumaphos) and renewed wax every 2–3 years. In contrast, Apiary 2 alternated Apistan (t-fluvalinate) and Apivar (amitraz), and did not renew wax annually. This likely favored the accumulation of these compounds or their degradation products in the colonies, threatening bee health [78]. Several studies indicate that exposure to sublethal concentrations of these compounds affects the bee immune system [2, 79, 80].

In this way, habitat, climate and *Varroa*-related factors may help explain the deterioration in health status and appearance of overt symptomatology in Apiaries 1 and 2. Indeed, Apiary 1 presented the lowest value of global quality (0.1). Interestingly, Apiary 2 presented the third lowest value (0.14), perhaps reflecting its high habitat quality (0.22) (Table 1).

Apiaries 3 and 4 had relatively low habitat quality (0.09) with respect to the other apiaries (Table 1). This likely reflects the lower orographic diversity and low elevation range (3a = 150 m; 4 = 190 m). In addition, an urbanized area next to Apiary 4 reduced the surface area of available resources for honey bee colonies. On the other hand, both locations had high landscape heterogeneity. The location around Apiary 3a had numerous patches (80) featuring 9 land cover types; 63 of the patches were patches of vegetation. The location around Apiary 4 also had numerous patches (67), 62 of which were patches of vegetation. Thus, the success of Apiaries 3a and 4 will likely depend on other factors such as climate and management.

Apiary 3 was moved to another location at a higher altitude (h = 1,055 m) in late spring as a result of the changing weather. The second location (3b) provided a better natural environment with a high elevation range (H7 = 490 m), absence of power lines and antennas (H6) and crops in the vicinity (H9). On the other hand, this second location lay on the edge of a large watercourse, which reduced the useable area within the total of 7.06 km^2^. Even so, habitat quality was higher than at the first location. Apiary 3 presented one of the highest health values in this study (0.27), reflecting better values for He1, He2 and He3, especially for *N*. *ceranae*, IAPV and—in contrast to Apiaries 1 and 2—DWV. The lower loads of DWV and IAPV may be related to the low *Varroa* load [70, 81], as Apiary 3 presented the lowest He4 of all six apiaries (mean of 126 mites per colony), and only one of ten colonies examined presented symptoms of varroosis in the second sampling. Other factors contributing to the high health status of Apiary 3 are the timely renewal of wax (all frames changed every 2–3 years), control of wax origin and use of ecological anti-*Varroa* treatment (oxalic acid). This apiary was rated as an intermediate alternative by AHP.

Apiary 4, located in a wooded area with high humidity and little sunshine, did not change its location during the season. Its health value was lower at the second sampling (0.06) than the first, and it ranked fifth at the end of the study period. This was probably because He1 worsened, primarily because of increases in loads of SBV and DWV, both of which are transmitted by *Varroa* [70, 82]. Indeed, this apiary contained abundant *Varroa* (mean of 1067 mites per colony) and showed symptoms in 8 of the 10 colonies studied. Ascospherosis was not observed, even though the excessive humidity and cold temperatures at location 4 are expected to promote it as previously described [83, 84]. This may reflect efficient management, such as frequent wax renewal, which still allowed high annual honey production (20 kg/colony). Nevertheless, AHP ranked this apiary among the worst alternatives (0.11).

Like Apiary 3, Apiary 5 was moved to a new, more favorable location at a higher altitude (1,150 m). As a result, the apiary was not exposed to the high temperatures (>35°C) and prolonged drought (>49 days without precipitation) that normally take place at the first location during the summer months. Extreme weather events such as storms, floods, and droughts negatively affect bees [4, 85, 86]. Changing the location of Apiary 5 also allowed the bees to take advantage of the different flowering periods. He1, He2 and He3 were lower at this second location, which had a health value of 0.11, placing it second among the apiaries in this respect. On the other hand, He4 was high: a mean of 1,023 mites per colony were present and varroosis symptoms were observed in 7 of the 9 colonies studied. It is possible that the anti-*Varroa* treatment used at that apiary (thymol) was inefficient, leaving higher-than-expected residual load of *Varroa* and associated viruses at the second sampling. The beekeeper at Apiary 5 controlled wax origin and limited its re-use. The combination of good habitat quality at high altitude as well as adequate management probably contributed to its excellent annual honey production (70 kg/colony). AHP ranked this apiary as one of the best alternatives (0.22).

The harsh climate conditions at Apiary 6, located at an altitude of 1,220 m, were attenuated by the humidity of a mountain stream close to the apiary and the surrounding oak forest containing scrub, pine, blackberry bush, wild rose and riverside vegetation with ash trees. This environment helped compensate for the scarce rainfall during the summer, which may help explain why the apiary showed a good health value (0.38) with low values of He1, He2 and He3. He4 was also probably low, since symptoms of mite presence were observed in only two of ten colonies during the second sampling, though mite abundance could not be quantified. Even though three acaricides were used at Apiary 5 to control *Varroa* (coumaphos, amitraz and t-fluvalinate), the apiary still showed a high health value, which together with high habitat quality (0.31) and adequate wax replacement and traceability, probably favored good health status, similar to the case of Apiary 5. Apiary 6 showed high annual honey production (28 kg/colony) and was ranked by AHP in first place among the six apiaries analyzed.

A deterioration in health status from the first to second sampling was observed at the two apiaries (1, 2) that were located throughout the year at altitudes <1,000 m with elevation ranges of only 100–200 m. In contrast, health factors improved from the first to second sampling when the apiary was located at higher altitude (Apiary 6, 1,220 m), or when the apiaries were moved to higher altitudes (Apiaries 3 and 5, from 800 to 1,100 m). Altitude was probably not the only explanation for the health improvement: locations around Apiaries 3b, 5 and 6 showed high environmental quality because of an elevation range of 450–490 m in the apiary area, which favored diverse vegetation cover.

One of our main concerns was the number of apiaries included in the final analysis, as we selected another 9 apiaries to have more reproducible results. However, we failed to obtain beekeepers’ permission to collect data about landscape heterogeneity, weather conditions, beehive management and health, so only habitat factors could be studied. However, we think that our results show important trends in the factors that should be prioritized in future studies. Our analysis of these six apiaries suggests that the combination of poor habitat quality, inadequate management, adverse climate conditions and health factors such as *Varroa* and pathogen loads may be decisive for the survival of colonies. These factors likely exert their negative effects mainly during summer-autumn, since most colony losses occur in winter [87–89]. Our results are consistent with research identifying pathogen load in summer-autumn as a predictor of winter losses [20] and colony strength [22]. Expert assessment of 39 possible causes of the decline in commercial honey bee colonies in the California almond industry highlighted two likely causes: the combination of *Varroa* mites and viruses, which reduce survival probability; and nutrient deficiency, which can cause population decline at the colony level and which by itself may be sufficient to explain large-scale colony collapse [90]. All apiaries in the present study showed the co-presence of *Varroa* and multiple viral infections. Further study is needed to examine whether and how this co-presence, together with environmental conditions, influences colony survival during winter. Perhaps the best way to ensure colony survival year-round is to take into account the ‘ecosystem health’ concept: an environment with sufficient habitat quality and various floral resources that bloom at non-overlapping times of the year.

Various factors in colony management may improve bee health status and honey productivity. Goulson et al. [4] proposed measures such as preventing competition between neighboring honeybee colonies, decreasing prophylactic use of aggressive anti-parasite and pathogen treatments, and avoiding fragmented habitats, which can ensure high flower diversity in the bee diet. Those authors [4] and others [33] also recommended various methods to reduce colony exposure to agrochemicals: extending distance to crops, shortening monofloral periods, evaluating synergistic effects between pesticides used inside and outside the hive, and implementing controls on the movement of all commercial bees. All these measures would help reduce chronic exposure of bees to numerous stressors, and they would improve the availability of nutritional resources. In these ways, such approaches might bolster bee resistance to disease, ensuring sustainable bee production.

The present study has highlighted some critical environmental factors that can support larger population of bees around the colony. When the apiary goal is honey production, the most important factors are a flower-rich field, diversity of patches of natural and seminatural areas, and absence of nearby crops to reduce exposure to pesticides. If the apiary purpose is pollination services, the most important factor is the reduction of extended pollination periods in these crops. Regional maps of habitat suitability may be useful for identifying good apiary locations for maximizing bee production, even before health problems appear. One strategy for reducing bee losses is to increase the influence of protective factors relative to the influence of stressors, but this must be done on a regional scale to be effective.

The analytical process used MCDA (AHP and weighted linear combinations) to evaluate all factors considered was selected with the kind of data herein and the aim of our analysis in mind, so that we could identify key factors for honey bee colonies, and classify colonies according to these key factors. Other models have been also used with different data sets. Remarkably, Convertino & Valverde 2013 [59] developed a Portfolio decision model (PDM) that integrated predictions by combining a MCDA with a Pareto optimization model to evaluate the importance of the factors and combinations between them in space and time. The model also included global sensitivity and uncertainty analyses.

Our study is mainly descriptive, we have evaluated several locations (each apiary) taking into account only data collected in the apiculture period 2014, namely we did not develop a temporal analysis. Also climatic data were collected during the apiculture period 2014, without presenting a great variability over that period. Thus, in the present study sensitivity analyses have not been included since we did not expect much variability in our data. However, we recommend carrying out a similar study considering a higher number of apiaries and years to develop a spatiotemporal model and to confirm the decision model, including in this case global sensitivity and uncertainly analyses.

## Conclusions

Although environmental evaluation of the six apiaries (eight locations) in the present study suggests that all locations are adequate, case-by-case evaluation using AHP identified several environmental, climate, management and health factors that may affect the ability of colonies to resist pathogens and maintain good health status. Long periods with scarce rainfall and excessive re-use of wax may reduce bee resistance to pathogens in apiaries showing low health status in late summer. Our study findings help lay the foundation for future work exploring these factors and their interactions in greater detail in order to guide strategies for maximizing bee and honey production.

## Supporting Information

S1 Table Description and evaluation of 9 factors used to evaluate Habitat quality. **Heterogeneous values with different scales**. H1: Number of land cover types; H2: Number of important vegetable species for bees; H3: Unfragmented area usable (km^2^); H4: Distance to permanent watercourses (m); H5: Distance to roads (m) (*primary roads; **secondary roads); H6: Distance high voltage power lines^+^, low voltage power lines^++^ and antennas^+++^ (m); H7: Difference in altitude (m); H8: Orographic diversity (km^2^); H9: Crop surface (km^2^).
(TIF)

S2 Table Description and evaluation of 9 factors used to evaluate Habitat quality to the additional apiaries. **Heterogeneous values with different scales**. H1: Number of land cover types; H2: Number of important vegetable species for bees; H3: Unfragmented area usable (km^2^); H4: Distance to permanent watercourses (m); H5: Distance to roads (m) (*primary roads; **secondary roads); H6: Distance high voltage power lines^+^, low voltage power lines^++^ and antennas^+++^ (m); H7: Difference in altitude (m); H8: Orographic diversity (km^2^); H9: Crop surface (km^2^).
(TIF)

S3 Table Description and evaluation of 4 factors used to evaluate Landscape heterogeneity. **Heterogeneous values with different scales**. L1: Total edge; L2: Mean patch size; L3: Number of patches; L4: Patch size standard deviation.
(TIF)

S4 Table Evaluation of climatic factors related to bee’s activity. PO = Poor conditions; AC = Adverse conditions; OC = Optimal conditions.
(TIF)

S5 Table Evaluation and development of beehive management factors. **Heterogeneous values with different scales**. M1: Annual honey production (kg/colony); M2: Number of honey produced types; M3: Anti-*Varroa* treatment; M4: N° of treatments/year; M6:% renewal of wax; M7: wax origins; M8: Movement of the apiary; M9: Presence of livestock.
(TIF)

S6 Table Characteristics and toxicological data of anti-*Varroa destructor* treatments used at the apiaries (M5). (0) Non-condition, (1) Low, (2) Moderate, (3) High. (*)N-2,4-dimethyl-phenyl-methylformanidine (more toxic and persistent) and 2,4-dimethyl-formanilide (na), 2,4 dimethylaniline mutagenic and carcinogenic (na). (**) Chlorferon; coumaphoxon, 6-hydroxyl-3-methylbenzofuran; Diethyl-3-acetoxy- phenylphosphorothioate. na = data not available.
(TIF)
