# Supplementary figures and images for: Importance of Ecological Factors and Colony Handling for Optimizing Health Status of Apiaries in Mediterranean Ecosystems
Source: PLoS One. 2016 Oct 11;11(10):e0164205. doi: 10.1371/journal.pone.0164205 (PMC5058545; doi:10.1371/journal.pone.0164205)

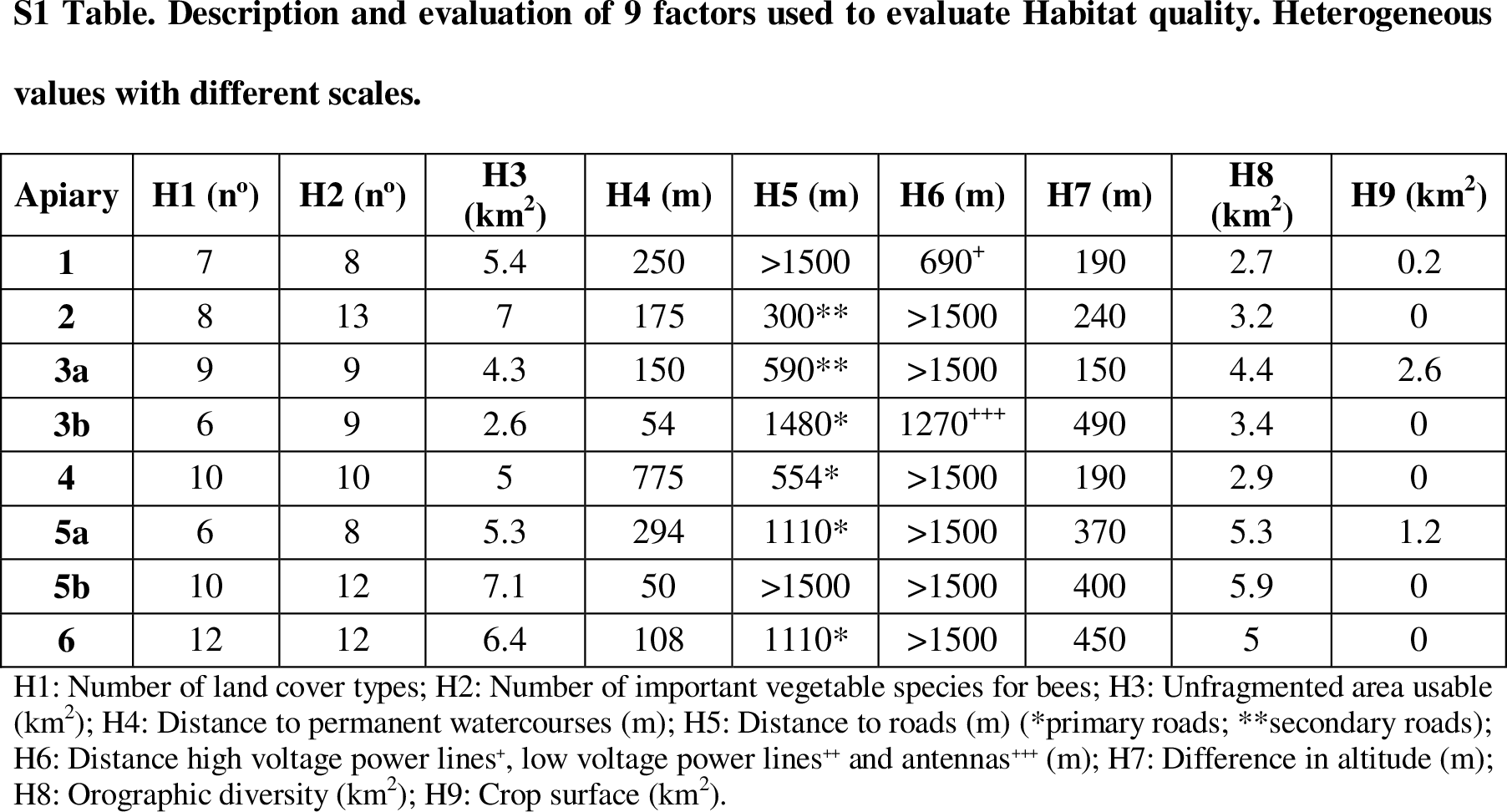

Supplement: S1 Table — Heterogeneous values with different scales. H1: Number of land cover types; H2: Number of important vegetable species for bees; H3: Unfragmented area usable (km2); H4: Distance to permanent watercourses (m); H5: Distance to roads (m) (*primary roads; **secondary roads); H6: Distance high voltage power lines+, low voltage power lines++ and antennas+++ (m); H7: Difference in altitude (m); H8: Orographic diversity (km2); H9: Crop surface (km2). (TIF) [file pone.0164205.s001.tif]

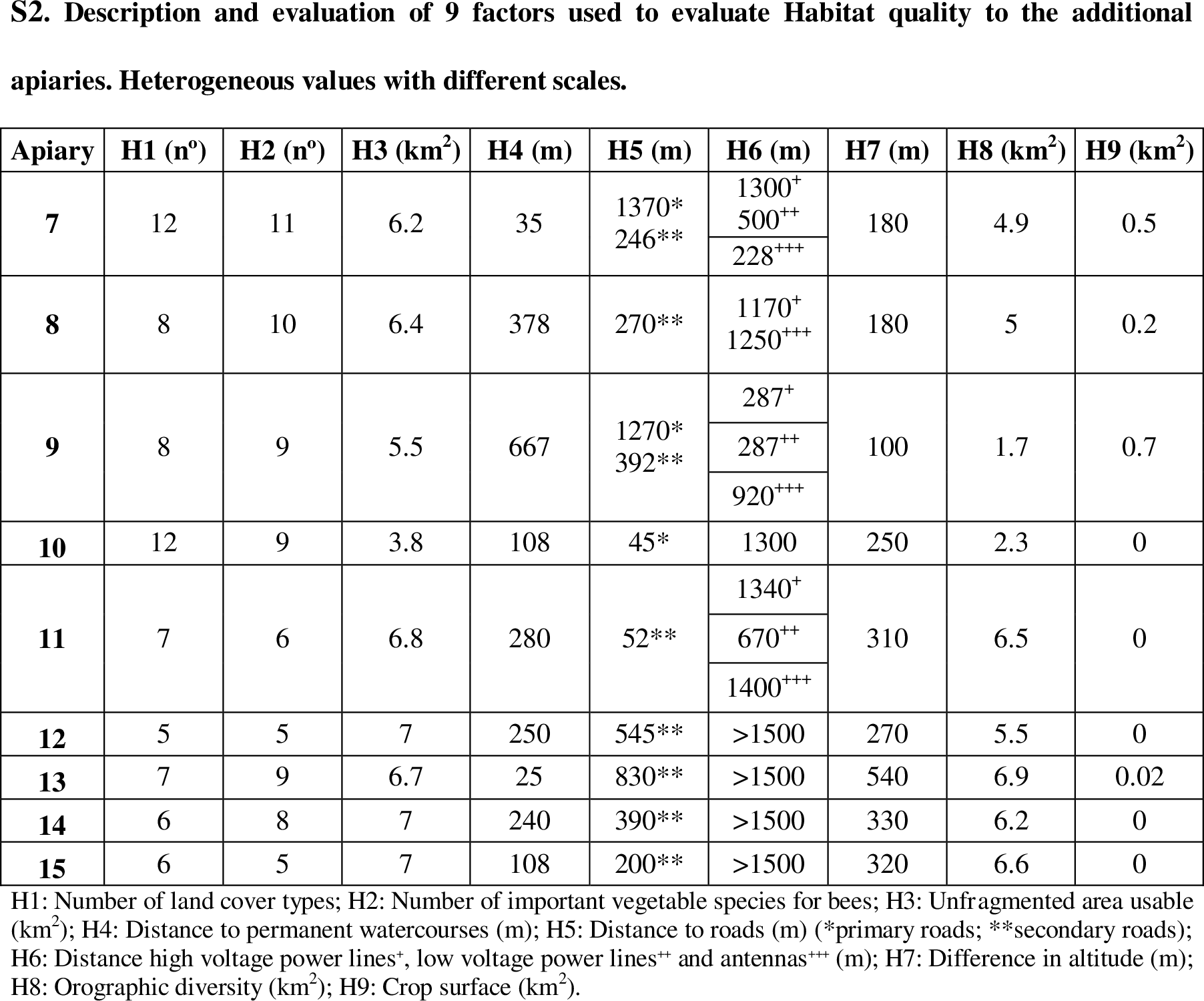

Supplement: S2 Table — Heterogeneous values with different scales. H1: Number of land cover types; H2: Number of important vegetable species for bees; H3: Unfragmented area usable (km2); H4: Distance to permanent watercourses (m); H5: Distance to roads (m) (*primary roads; **secondary roads); H6: Distance high voltage power lines+, low voltage power lines++ and antennas+++ (m); H7: Difference in altitude (m); H8: Orographic diversity (km2); H9: Crop surface (km2). (TIF) [file pone.0164205.s002.tif]

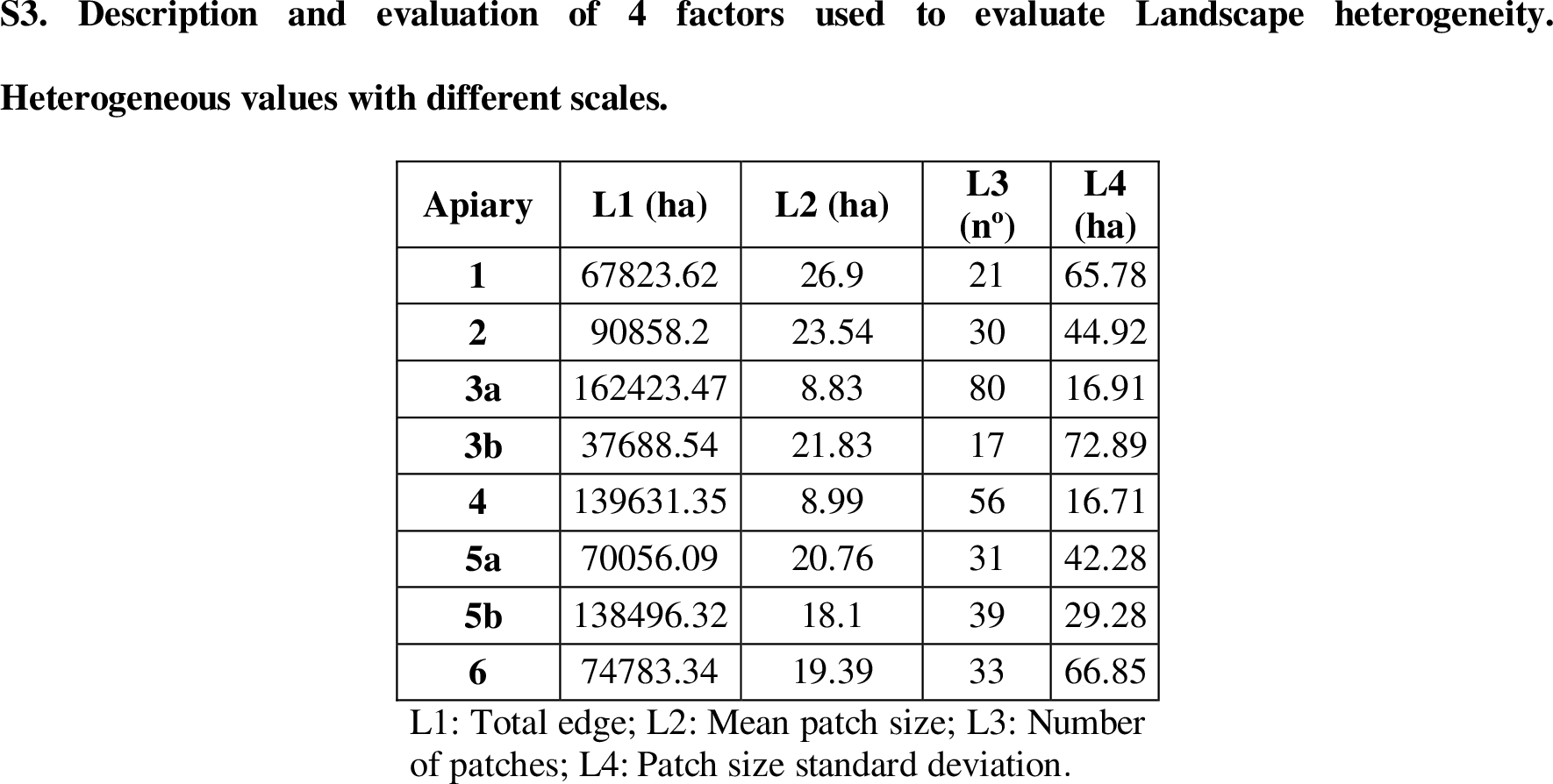

Supplement: S3 Table — Heterogeneous values with different scales. L1: Total edge; L2: Mean patch size; L3: Number of patches; L4: Patch size standard deviation. (TIF) [file pone.0164205.s003.tif]

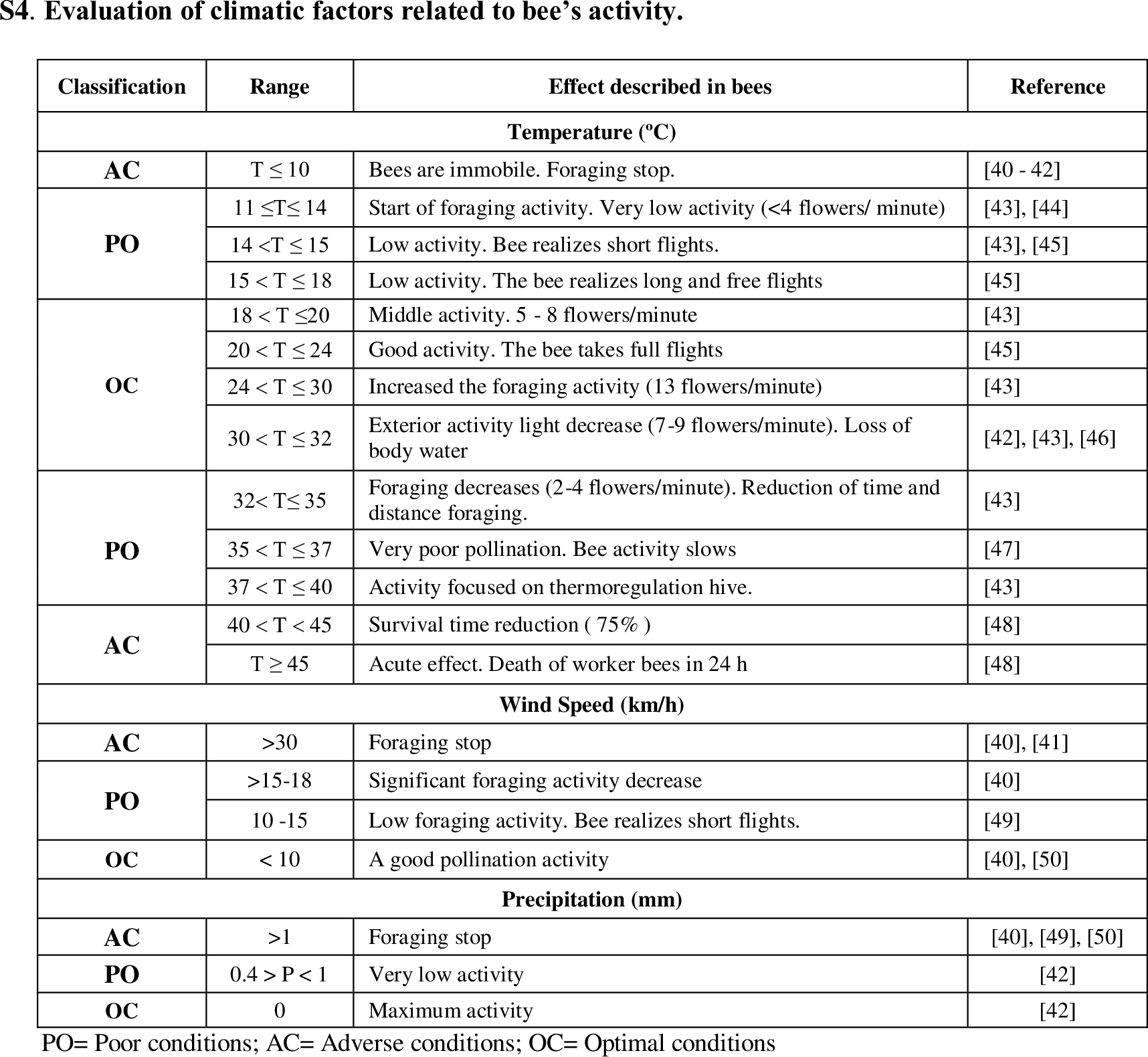

Supplement: S4 Table — PO = Poor conditions; AC = Adverse conditions; OC = Optimal conditions. (TIF) [file pone.0164205.s004.tif]

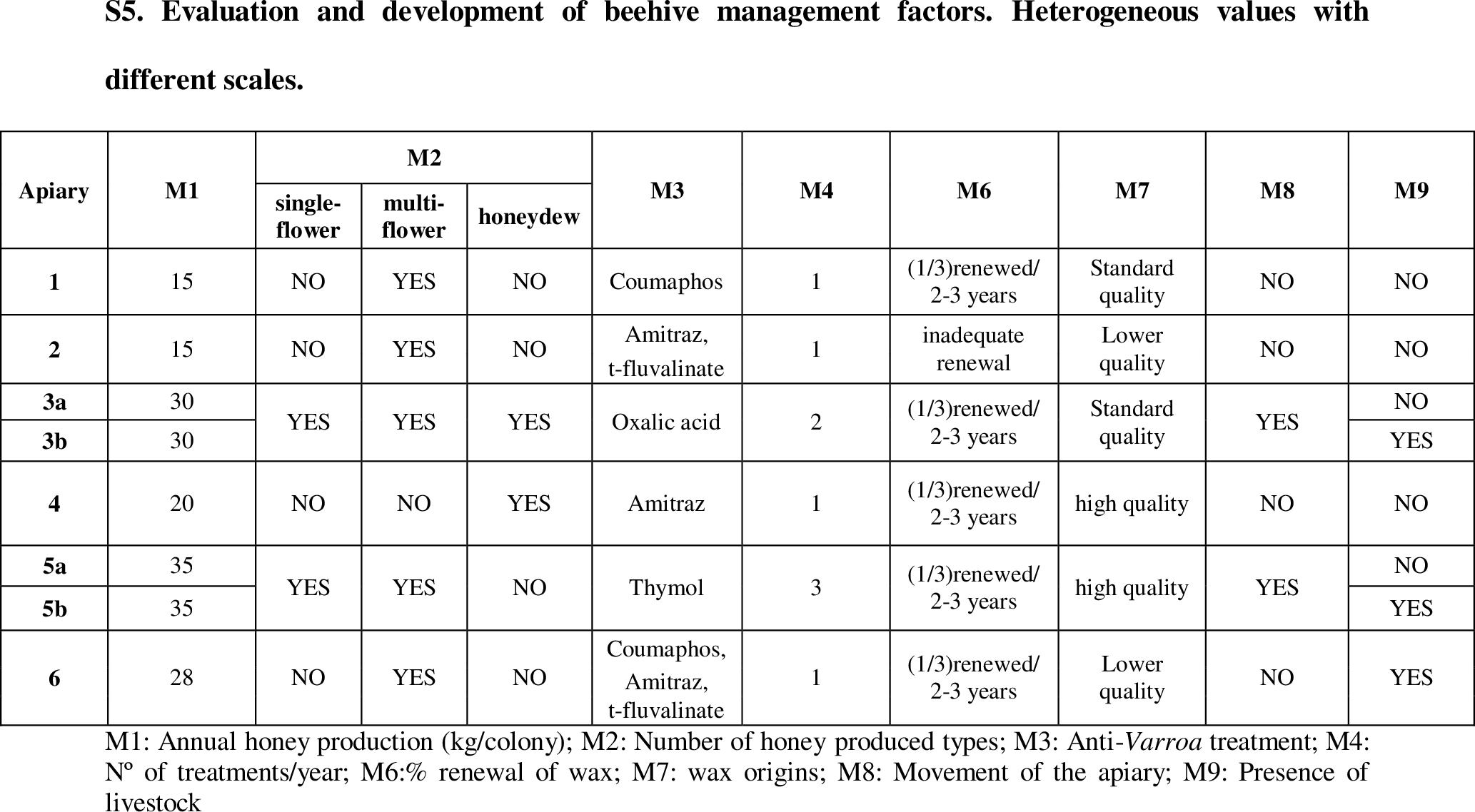

Supplement: S5 Table — Heterogeneous values with different scales. M1: Annual honey production (kg/colony); M2: Number of honey produced types; M3: Anti-Varroa treatment; M4: N° of treatments/year; M6:% renewal of wax; M7: wax origins; M8: Movement of the apiary; M9: Presence of livestock. (TIF) [file pone.0164205.s005.tif]

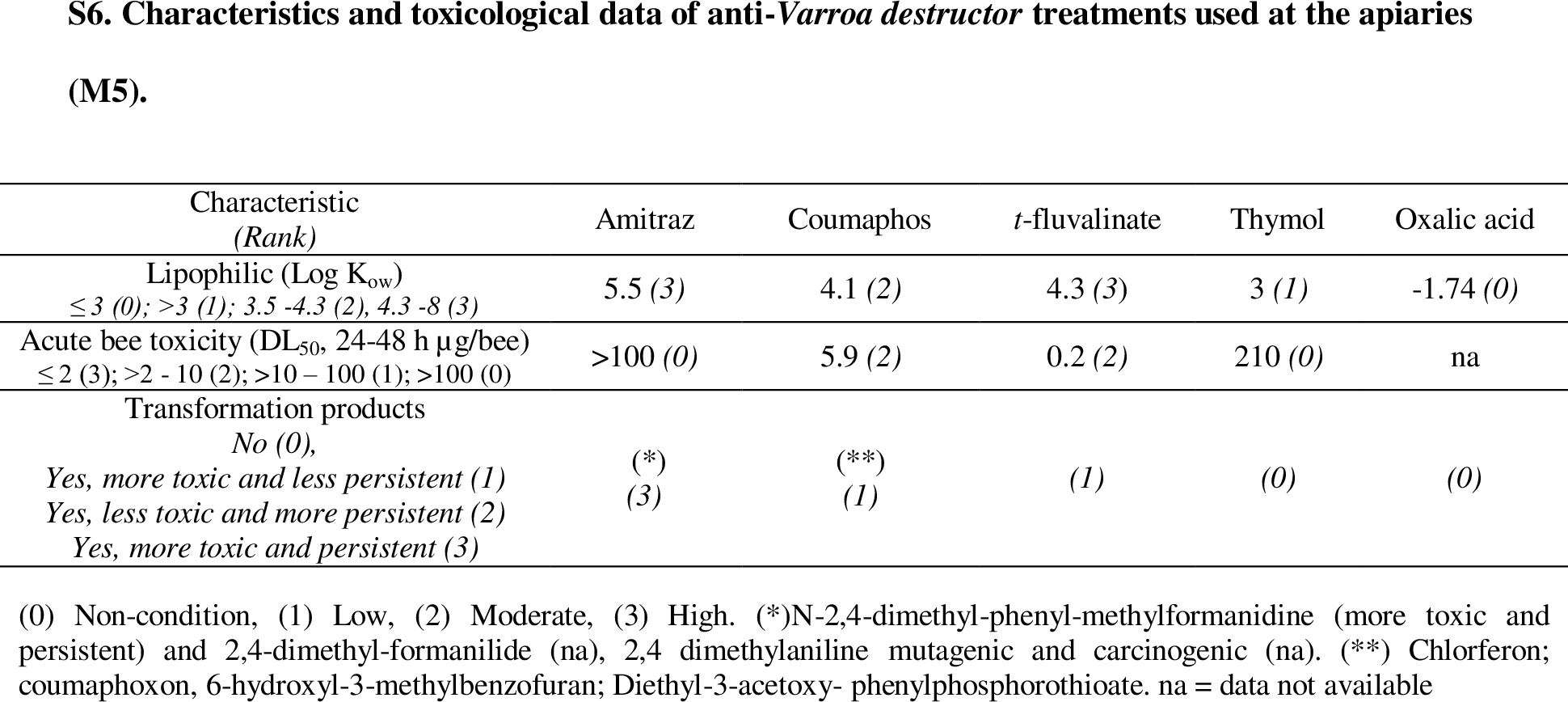

Supplement: S6 Table — (0) Non-condition, (1) Low, (2) Moderate, (3) High. (*)N-2,4-dimethyl-phenyl-methylformanidine (more toxic and persistent) and 2,4-dimethyl-formanilide (na), 2,4 dimethylaniline mutagenic and carcinogenic (na). (**) Chlorferon; coumaphoxon, 6-hydroxyl-3-methylbenzofuran; Diethyl-3-acetoxy- phenylphosphorothioate. na = data not available. (TIF) [file pone.0164205.s006.tif]
